# Pre-analytical reporting in AI-assisted cervical cytology: a scoping review of data acquisition documentation

**DOI:** 10.3389/frai.2026.1884193

**Published:** 2026-07-09

**Authors:** Alina Elena Sultana, Răzvan George Condorovici, Ştefana Duţă, Andra Laura Dobre, Corina Elena Petean

**Affiliations:** Department of Applied Electronics and Information Engineering, National University of Science and Technology POLITEHNICA Bucharest, Bucharest, Romania

**Keywords:** artificial intelligence, cervical cytology, digital pathology, pre-analytical variability, PRECY-AI, reporting quality, reproducibility, scoping review

## Abstract

Artificial intelligence (AI) models for cervical cytology screening have achieved pooled accuracy and sensitivity values exceeding 90% in recent meta-analyses, and several commercial systems are now in clinical use. However, whether these results generalize across laboratories, scanners, and clinical settings depends on pre-analytical factors—sample preparation, staining, digitization, and annotation—that are known to introduce substantial variability into the data that models consume. This scoping review assessed how consistently these factors are documented in the cervical cytology AI literature. We examined 28 datasets published between 2005 and 2025, extracting information on 16 pre-analytical variables spanning sample preparation, digitization, and annotation. The mean reporting completeness was 11.4 out of 16 variables (71.2%). Digitization was the weakest category (mean 60.2%), with scanning mode unreported in 57.1% of datasets, image file format in 60.7%, and color normalization status in 78.6%. Staining protocol was mentioned by 75.0% of datasets but described in sufficient procedural detail by only 7.1%. Quantitative inter-annotator agreement was provided by 14.3% of datasets, despite well-documented inter-observer variability in cervical cytology. Notably, the variables with the lowest reporting rates correspond to those identified in the digital pathology literature as the most significant sources of AI model performance variability. To address this gap, we propose PRECY-AI (Pre-analytical Reporting Checklist for Cervical Cytology AI), a 16-item checklist of essential and recommended reporting items designed to complement existing general-purpose guidelines such as TRIPOD+AI and CLAIM. Adoption of domain-specific pre-analytical reporting standards could improve the reproducibility, comparability, and clinical translatability of cervical cytology AI research.

## 1 Introduction

Cervical cancer remains one of the most significant threats to women's health worldwide. In 2022, approximately 660,000 new cases were diagnosed globally, resulting in roughly 350,000 deaths, with over 85% of this burden falling on low- and middle-income countries (LMICs) (Sung et al., 2021; Mbelwa et al., 2026). The disease is largely preventable through vaccination against the human papillomavirus (HPV) and through systematic screening, which can detect precancerous lesions before they progress to invasive cancer. Cervical cytology—whether through conventional Papanicolaou (Pap) smears or liquid-based cytology (LBC) preparations such as ThinPrep and SurePath—has been a cornerstone of these screening efforts for decades (Swailes et al., 2019). However, the manual review of cytology slides is labor-intensive, subject to inter-observer variability, and heavily dependent on the expertise of cytotechnologists and cytopathologists, whose availability remains critically limited in many regions (Nanda et al., 2000; Koonmee et al., 2017).

Over the past decade, artificial intelligence (AI) has emerged as a promising tool for addressing these limitations. Deep learning models, particularly convolutional neural networks (CNNs), have demonstrated remarkable performance in detecting and classifying cervical cell abnormalities from cytology images. A recent meta-analysis by (Liu et al., 2025) estimated pooled accuracy values of 94% for AI-assisted Pap smear analysis and 90% for AI-assisted ThinPrep cytologic test (TCT), with sensitivity exceeding 95% in both modalities. The methodological landscape has also expanded considerably: a systematic review by Mbelwa et al. (2026) cataloged 96 studies published between 2014 and 2025, documenting the progression from traditional machine learning classifiers to CNNs, Vision Transformers, generative adversarial networks, and multimodal fusion models that combine imaging data with clinical text. Concurrently, several AI-based solutions for cervical cytology screening have reached commercial availability. Chong and Bychkov (2025) identified at least eight commercial systems currently on the international market, including the Hologic Genius Digital Diagnostic System, which received U.S. Food and Drug Administration (FDA) approval in 2024, and systems from Datexim, CellSolutions, Techcyte, Landing Med, and KFBio, among others.

These developments collectively suggest that the field has matured substantially in terms of both the diversity of AI methods applied and the performance levels achieved. The questions of *what models have been used* and *how well they perform* have been addressed by multiple comprehensive reviews and quantitative syntheses. However, a critical dimension remains largely unexplored: *under what data acquisition conditions were these results obtained?*

The path from a patient's cervical sample to an AI model's prediction traverses a multi-step pipeline in which variability can be introduced at every stage. The choice of cytology preparation method determines fundamental image characteristics—conventional smears produce heterogeneous, overlapping cell distributions, whereas LBC methods yield thinner, more uniform layers, with ThinPrep and SurePath each producing distinct morphological signatures (Leung et al., 1997). The Papanicolaou staining protocol, a polychromatic technique involving five dyes, is known to exhibit considerable variation across laboratories due to differences in reagent concentrations, immersion durations, and even the pH of local tap water (Gill et al., 1974). At the digitization stage, whole-slide image (WSI) scanners differ in their optical characteristics—including numerical aperture, pixel resolution, and focusing methodology—and models trained on images from one scanner have been shown to suffer significant performance degradation when tested on images from another, a phenomenon referred to as scanner-induced domain shift (Howard et al., 2021). In cytology specifically, the three-dimensional structure of cell clusters means that scanning mode (single plane, z-stack, extended depth of focus, or volumetric scanning) determines which cellular features are captured and which are lost (Chong and Bychkov, 2025; Patel et al., 2021). Finally, the annotation process introduces its own variability: the number and qualification of annotators, the classification system employed (the Bethesda System, Pap classes, or CIN grading), and whether ground truth is confirmed by histopathology or based on a single expert's cytological reading all shape the labels against which models are trained and evaluated (Stoler and Schiffman, 2001).

Despite the well-documented influence of these pre-analytical factors, existing reviews of AI in cervical cytology have primarily extracted and compared information about model architectures and reported performance metrics. When (Liu et al., 2025) conducted regression analyses to identify sources of heterogeneity in their meta-analysis, they found that sample size and ground truth definition were significant contributors—but acquisition-related variables such as preparation type, scanner model, and scanning mode were not available for analysis, precisely because they are inconsistently reported across studies. Similarly, while Chong and Bychkov (2025) documented that the real-world performance of commercial AI systems can deviate substantially from manufacturer specifications—citing a case where vendor-claimed specificity of ≥63.5% dropped to 12% in independent validation—they attributed this in part to differences in acquisition context (LBC type, scanner, staining) that are rarely controlled or reported in the research literature.

This gap has practical consequences at multiple levels. For researchers, the absence of standardized reporting of acquisition conditions makes it impossible to determine whether performance differences across studies reflect genuine advances in model architecture or merely differences in the underlying data. For clinicians and policymakers considering the adoption of AI screening tools—particularly in LMICs where acquisition conditions may differ substantially from those used in model development—studies that omit these details provide weak evidence for deployment decisions. For the field as a whole, the inability to account for pre-analytical variability limits the interpretability of cross-study comparisons and meta-analyses, and undermines efforts toward reproducibility.

This scoping review aims to address this gap by systematically assessing the extent and quality of pre-analytical reporting across cervical cytology datasets used in AI research. Our unit of analysis is the dataset—whether a publicly available benchmark or a private institutional collection—rather than the individual AI study, because it is the dataset description that determines whether acquisition conditions are documented at the source. We examined 28 datasets spanning 2005 to 2025, extracting information on 16 pre-analytical variables covering sample preparation, digitization, and annotation. Rather than cataloging model architectures or comparing performance metrics—tasks already accomplished by recent reviews (Mbelwa et al., 2026; Liu et al., 2025; Chong and Bychkov, 2025)—we focus on a complementary question: *do published datasets provide sufficient information about how their image data was acquired, prepared, digitized, and annotated to enable reproducibility, cross-study comparison, and informed clinical deployment?* As a secondary contribution, we identify the most common reporting gaps and propose PRECY-AI (Pre-analytical Reporting Checklist for Cervical Cytology AI), a 16-item checklist designed to complement existing general-purpose reporting guidelines such as TRIPOD+AI (Collins et al., 2024) and CLAIM (Mongan et al., 2020).

## 2 Materials and methods

### 2.1 Sources of pre-analytical variability in cervical cytology AI

**Figure 1:**
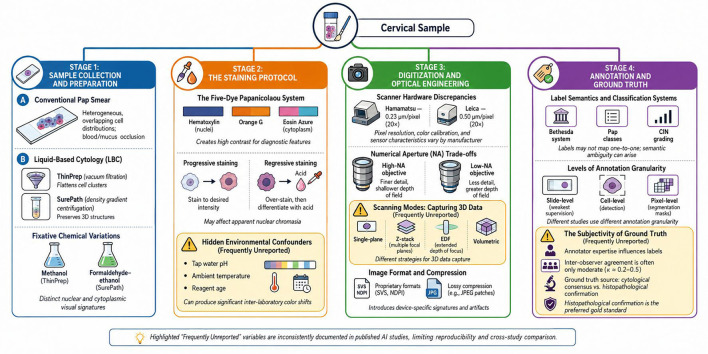
Overview of the cervical cytology data acquisition pipeline and sources of variability at each stage. The pipeline spans four stages: (1) sample collection and preparation, where the choice between conventional Pap smear and liquid-based cytology (ThinPrep vs. SurePath) determines cell distribution, overlap, and fixative chemistry; (2) the Papanicolaou staining protocol, a five-dye polychromatic system in which protocol variant (progressive vs. regressive), reagent conditions, and environmental factors produce inter-laboratory color variation; (3) digitization, where scanner hardware (numerical aperture, pixel resolution, color calibration), scanning mode (single-plane, z-stack, extended depth of focus, or volumetric), and image format introduce device-specific signatures; and (4) annotation and ground truth, where the classification system, annotation granularity, annotator expertise, and the basis for ground truth (cytological consensus vs. histopathological confirmation) shape the labels against which AI models are trained and evaluated. Elements marked as “Frequently Unreported” indicate variables that, based on the present review, are inconsistently documented in published AI studies, limiting reproducibility and cross-study comparison.

Before a deep learning model processes a single pixel from a cervical cytology image, the underlying biological sample has passed through a multi-step pipeline in which variability is introduced at every stage. For the computer scientist, understanding these sources of variability is essential: they determine the statistical properties of the input data, and they explain why a model achieving 98% accuracy on one dataset may fail when deployed in a different clinical setting. This section describes the major stages of this pipeline—sample collection and preparation, staining, digitization, and annotation—and explains how each one shapes the data that AI models ultimately consume. Figure 1 provides a schematic overview of the full acquisition-to-inference pipeline and the sources of variability at each stage.

#### 2.1.1 Sample collection and preparation

Cervical cytology begins with the physical collection of cells from the transformation zone of the cervix—the anatomical region where the squamous epithelium of the ectocervix meets the columnar epithelium of the endocervical canal, and where nearly all cervical intraepithelial neoplasia and invasive carcinomas originate (Sellors and Sankaranarayanan, 2003). A clinician uses a spatula or brush to scrape cellular material from this region, and the manner in which these cells are subsequently transferred to a glass slide determines the fundamental visual characteristics of the resulting image.

In the conventional Papanicolaou (Pap) smear, collected cells are smeared directly onto a glass slide. Conventional Pap smears remain widely used globally due to their cost-effectiveness and lack of requirement for specialized liquid-based processing equipment, making them particularly accessible in low-resource settings. However, this method produces a heterogeneous distribution of cells, with frequent clumping, overlapping layers, and obscuring material such as blood and mucus (Bengtsson, 1999; William et al., 2018). For a convolutional neural network, this means the input images contain high levels of occlusion, variable cell density, and non-uniform backgrounds—all of which increase the difficulty of both cell-level and slide-level classification tasks.

Modern laboratories have largely transitioned to liquid-based cytology (LBC), in which the collected cells are rinsed into a vial of preservative solution and then mechanically processed to create a thinner, more uniform layer on the slide. The two dominant commercial LBC systems—ThinPrep (Hologic) and SurePath (BD Diagnostics)—employ fundamentally different processing principles. ThinPrep uses vacuum filtration through a polycarbonate membrane to deposit cells in a circular monolayer, whereas SurePath uses density gradient centrifugation to separate and layer cells (Chong and Bychkov, 2025). These mechanical differences produce distinct morphological signatures that are visible to both human observers and machine learning models: ThinPrep tends to flatten cell clusters and fragment them, while SurePath preserves larger, more three-dimensional cluster configurations (Lee et al., 2024). The preservative solutions themselves also differ—ThinPrep uses methanol, whereas SurePath uses a formaldehyde-ethanol mixture—leading to different fixation characteristics that affect nuclear and cytoplasmic appearance (Rebolj et al., 2015).

The practical consequence for AI is that a model trained on images from one LBC system may not generalize to the other. (Welch et al., 2024) demonstrated this directly: deep learning models trained on SurePath multicellular images achieved over 90% accuracy when tested on other SurePath images, but accuracy dropped to as low as 60.29% when the same models were tested on ThinPrep images, even after applying domain adaptation techniques. This finding underscores why reporting the preparation method is not merely a methodological formality—it is a critical determinant of whether reported performance metrics can be expected to transfer to a different laboratory's workflow.

#### 2.1.2 Staining

Once cells are deposited on a glass slide, they must be stained to make cellular structures visible under a microscope. While hematoxylin-only staining provides clear nuclear detail, it lacks the cytoplasmic contrast essential for identifying metabolic cellular changes, which is why the full Papanicolaou polychromatic protocol remains the clinical standard. The Papanicolaou stain is a polychromatic technique that uses five dyes in three sequential solutions: hematoxylin stains the cell nucleus blue-violet, Orange G (OG-6) stains keratin and mature squamous cells orange, and a mixture of Eosin Azure (EA) stains the cytoplasm in shades of pink, green, and blue depending on cell maturity and metabolic state (Gill et al., 1974; Koss and Melamed, 2006). The objective is to achieve high nuclear-to-cytoplasmic contrast, which is the primary visual feature used by both cytopathologists and computer vision models to distinguish normal from abnormal cells.

However, the color balance of a Pap-stained slide is rarely uniform across laboratories. The staining protocol exists in two major variants—progressive and regressive—which produce different color profiles. In the progressive method (using Gill's or Mayer's hematoxylin), the tissue is stained to the desired intensity and then blued. In the regressive method (using Harris hematoxylin), the tissue is intentionally overstained and then selectively de-stained with acid alcohol, a step that requires precise timing and can lead to hyper- or hypochromatic nuclei if poorly controlled (Gruber et al., 2002). Beyond these protocol-level differences, environmental factors such as the pH of the water used for bluing, the ambient temperature, the age of the reagent solutions, and the duration of immersion in each dye bath all contribute to inter-laboratory color variation (Clarke and Treanor, 2017).

For machine learning, this variability is particularly problematic because standard RGB imaging systems have only three channels to capture the combined absorption spectra of five dyes (Takeyama et al., 2025). This creates an underdetermined decomposition problem: different combinations of biological state and staining intensity can map to the same RGB values, and conversely, the same biological structure can appear in visually different colors depending on the laboratory's staining protocol. Computational approaches to this problem include stain normalization—methods such as Macenko decomposition, Reinhard color transfer, and GAN-based style transfer that attempt to standardize the color distribution of input images to match a reference template (Macenko et al., 2009; Tellez et al., 2019). While these techniques can partially mitigate staining variability, they add a preprocessing step that itself introduces potential artifacts, and their effectiveness varies across tissue and cell types (Khan et al., 2026).

From a reporting perspective, the implication is clear: a study that does not specify its staining protocol, or that uses a public dataset without documenting the staining conditions under which the images were acquired, leaves the reader unable to assess whether its reported performance metrics would hold under different staining conditions.

#### 2.1.3 Digitization

The transition from a physical glass slide to a digital image introduces a second tier of technical variability, often referred to in the digital pathology literature as scanner-induced domain shift (Howard et al., 2021). The hardware and software used for digitization determine the optical resolution, color rendition, and depth-of-field characteristics of the resulting image, all of which directly affect what a neural network can learn from the data.

##### 2.1.3.1 Scanner hardware characteristics

Whole-slide image (WSI) scanners differ in several key technical parameters. The numerical aperture (NA) of the objective lens determines both the resolving power and the depth of field: a higher NA captures finer chromatin texture—essential for grading nuclear atypia—but produces a shallower focal plane, making the system more sensitive to focus artifacts (Patel et al., 2021). The pixel resolution at a given magnification varies across manufacturers (for example, approximately 0.50 μ m/pixel for Leica Aperio AT2 at 20 × equivalent vs. 0.23 μ m/pixel for Hamamatsu NanoZoomer S360), meaning that identically-sized biological structures occupy different numbers of pixels depending on the scanner used (Ardon et al., 2025). Color calibration also varies: each scanner's sensor, illumination source, and white-balance algorithm produce a device-specific color profile that can cause identical staining to appear differently in the digital image (Genis et al., 2021).

##### 2.1.3.2 Scanning mode and the challenge of three-dimensionality

Unlike histological tissue sections, which are typically 3–5 μ m thick and can be adequately captured in a single focal plane, cytology preparations present cells and cell clusters that are thicker and often multi-layered (Chong and Bychkov, 2025). This makes the choice of scanning mode a critical variable for cytology AI:

- Single-plane scanning captures one focal layer, which is standard in histopathology but inadequate for cytology, as cells above or below the focal plane appear blurred and their nuclear detail—the primary diagnostic feature—is lost.
- Z-stack scanning captures multiple focal planes at defined intervals above and below the estimated optimal focus, preserving three-dimensional information but producing substantially larger files (typically 5–15 × the size of a single-plane scan).
- Extended depth of focus (EDF) computationally fuses multiple z-planes into a single all-in-focus image, reducing file size but potentially introducing fusion artifacts.
- Volumetric scanning, introduced by Hologic in the Genius Digital Diagnostic System, uses an oblique light source to generate single-focus images from 14 focal planes, offering a compromise between depth capture and scan time (Chong and Bychkov, 2025).

The choice among these modes directly affects the information content of the resulting image. A model trained on z-stacked images may rely on features that are absent from single-plane scans; conversely, EDF artifacts may introduce patterns that a model could learn as spurious correlations.

##### 2.1.3.3 Microscope-captured images vs. whole-slide images

A substantial number of AI studies in cervical cytology do not use WSI scanners at all. Instead, they capture images through a conventional microscope equipped with a digital camera—a setup that is common in resource-limited settings and in studies that use older public datasets such as Herlev (captured with a specialized microscope and frame grabber at 0.201 μ m/pixel resolution) (Mbelwa et al., 2026). Microscope-captured images differ from WSI in field of view, resolution uniformity, illumination consistency, and the absence of automated focusing. These differences are rarely discussed in AI studies but represent a fundamental data modality distinction that affects model transferability.

##### 2.1.3.4 Image format and compression

WSI scanners produce images in proprietary formats—SVS (Leica), NDPI (Hamamatsu), MRXS (3DHistech)—each with different compression schemes and tiling structures. Many AI studies extract patches from these formats and save them as JPEG or PNG files, introducing lossy compression artifacts that vary depending on the quality parameter used. While the adoption of the DICOM standard for digital pathology (Supplement 145) is beginning to address interoperability (Herrmann et al., 2018), the vast majority of published AI studies predate widespread DICOM adoption and use heterogeneous file formats.

The cumulative effect of these digitization variables is that two digital images of the same physical slide, scanned on different devices, can differ substantially in their pixel-level characteristics. Howard et al. (2021) demonstrated that deep learning models can learn to distinguish images by their site of origin—effectively learning scanner signatures rather than biological features—and that this site-specific information can act as a confounding variable that inflates apparent model performance when training and test data share the same acquisition setup.

#### 2.1.4 Annotation and ground truth

The final stage of the data acquisition pipeline—and arguably the one with the greatest impact on what a model learns—is the creation of labels against which the model is trained and evaluated.

##### 2.1.4.1 Classification systems and label semantics

Cervical cytology findings are reported using one of several classification systems, each with different granularity and terminology. The Bethesda System (TBS), now in its third edition (Nayar and Wilbur, 2015), is the most widely adopted and classifies findings into categories including Negative for Intraepithelial Lesion or Malignancy (NILM), Atypical Squamous Cells of Undetermined Significance (ASC-US), Low-Grade Squamous Intraepithelial Lesion (LSIL), Atypical Squamous Cells—Cannot Exclude HSIL (ASC-H), High-Grade Squamous Intraepithelial Lesion (HSIL), and Squamous Cell Carcinoma (SCC). Older systems, including the original Pap classification (Classes I–V) and the Cervical Intraepithelial Neoplasia (CIN) grading system, remain in use in some studies and datasets. As (Mbelwa et al., 2026) note, some AI studies convert between these systems (e.g., mapping LSIL to CIN1 and HSIL to CIN2/3), but this mapping is not always one-to-one, introducing label ambiguity. For machine learning, this means that nominally similar multi-class classification tasks may actually have different label semantics across studies, complicating cross-study performance comparison.

##### 2.1.4.2 Annotation granularity

AI studies in cervical cytology operate at different levels of annotation granularity. Slide-level labels (e.g., “this slide is HSIL”) provide the weakest supervision but are the easiest to obtain from clinical workflows. Cell-level labels require an expert to identify and classify individual cells, which is more informative but orders of magnitude more labor-intensive. Pixel-level annotations (segmentation masks for nucleus, cytoplasm, and background) provide the richest spatial information but are the most costly to produce and are available in only a few datasets, such as the ISBI 2014 and 2015 challenge sets (Mbelwa et al., 2026). The annotation granularity determines which types of models can be trained (e.g., whole-slide classifiers vs. cell detection models vs. segmentation networks) and affects reported metrics in ways that are not always comparable across studies.

##### 2.1.4.3 Annotator expertise and inter-observer variability

Cervical cytology is known to have substantial inter-observer variability. Stoler and Schiffman (2001), in the ASCUS-LSIL Triage Study, demonstrated that even among experienced cytopathologists, the agreement on specific TBS categories is moderate at best, with kappa values typically ranging from 0.2 to 0.5 for borderline categories such as ASC-US and LSIL. This means that the “correct” label for a given cell or slide is often itself uncertain. A model trained on annotations from a single pathologist may learn that pathologist's diagnostic tendencies—their systematic biases and threshold preferences—rather than an objective representation of disease state. (Chong and Bychkov, 2025) recommend that, for AI validation, the ground truth should ideally be established through histopathological confirmation rather than cytological consensus, precisely because cytology-based labels carry this inherent uncertainty.

##### 2.1.4.4 The ground truth problem

The strongest form of ground truth in cervical screening is histopathological confirmation from a biopsy: the patient's tissue is examined histologically, and the diagnosis is mapped back to the corresponding cytology slide. However, most AI studies do not have access to matched histopathology. Many rely on a single expert's cytological reading as ground truth, and studies that use public datasets often inherit whatever labels the dataset provides without documenting or questioning the process by which those labels were created. The Herlev dataset, for example, was annotated by cytopathologists at Herlev University Hospital in Denmark (Mbelwa et al., 2026), but the specifics of how many annotators were involved, whether disagreements were resolved by consensus, and whether any inter-annotator agreement metrics were computed are not consistently reported in the studies that use it.

#### 2.1.5 Dataset provenance and benchmark limitations

Public benchmark datasets play a central role in AI research by enabling reproducibility and cross-method comparison. In cervical cytology, a small number of datasets dominate the literature: Herlev (917 Pap smear images), SIPaKMeD (4,049 single cell images), CRIC (400 slide images with 11,534 classified cells), HiCervix (40,229 annotated cell images from 4,496 whole-slide images), and the ISBI 2014/2015 segmentation challenge datasets (Mbelwa et al., 2026). These datasets vary substantially in their documentation of acquisition conditions. SIPaKMeD specifies the microscope model (Olympus BX53F with CCD camera) and cell classification categories but provides limited information on staining protocol. Herlev reports the microscope resolution (0.201 μ m/pixel) and includes manual segmentation but does not detail staining conditions. CRIC documents the microscope (Zeiss AxioImager), camera, magnification, and resolution, providing one of the more complete acquisition descriptions among public cytology datasets.

The critical issue is that when a study reports, for example, 98% accuracy on SIPaKMeD, this result is entangled with every acquisition decision that produced that specific dataset—the microscope, the staining, the cell cropping procedure, the annotator pool. Without knowing these conditions, and without understanding how they compare to the conditions in a target deployment setting, the 98% figure provides limited evidence for clinical utility. Two studies achieving similar accuracy on the same benchmark tell us primarily about their relative model architectures; they tell us almost nothing about how either model would perform in a different laboratory.

### 2.2 Methodology

This scoping review follows the Preferred Reporting Items for Systematic Reviews and Meta-Analyses extension for Scoping Reviews (PRISMA-ScR) guidelines (Tricco et al., 2018).

#### 2.2.1 Research questions

The review was guided by three research questions:

RQ1 What proportion of cervical cytology datasets used in AI research report key pre-analytical variables related to sample preparation, digitization, and annotation?
RQ2 How does reporting completeness vary by dataset characteristics, including year of publication, geographic origin, and dataset source (public vs. private)?
RQ3 Which pre-analytical variables are most frequently unreported, and how do these gaps relate to known sources of variability affecting AI model performance?

#### 2.2.2 Eligibility criteria

We included datasets meeting all of the following criteria: (1) used for developing or evaluating AI or machine learning models for cervical cytology image analysis (classification, detection, or segmentation); (2) based on Pap smear or liquid-based cytology images; (3) described in a publication reporting quantitative performance metrics; (4) published between 2005 and 2025; and (5) described in English. We excluded datasets used exclusively for colposcopy, histopathology, or HPV typing; datasets described only in abstracts, editorials, or letters; and datasets for which no image-level description was available.

#### 2.2.3 Search strategy and study selection

Candidate datasets were identified through two complementary approaches. First, we systematically extracted all datasets referenced in three recent comprehensive reviews of AI in cervical cytology: (Mbelwa et al., 2026) (96 studies, 2014–2025), (Liu et al., 2025) (51 cytology studies, 2012–2024), and (Chong and Bychkov, 2025) (commercial systems review, 2025). These reviews collectively cover the Scopus, PubMed, IEEE Xplore, Embase, Cochrane Library, and Google Scholar databases. Second, we performed supplementary searches in PubMed and Google Scholar using the terms *(“cervical cytology” OR “Pap smear” OR “liquid-based cytology”) AND (“dataset” OR “benchmark” OR “image database”)* to identify dataset descriptor publications not captured by the existing reviews. Datasets were screened against the eligibility criteria by two reviewers independently, with disagreements resolved by consensus.

#### 2.2.4 Data extraction

For each included dataset, we extracted information across six categories: study metadata, sample preparation, digitization, annotation, dataset characteristics, and model performance (the latter recorded minimally for contextualization only). The extraction was organized around 16 key pre-analytical variables spanning three categories: sample preparation (cytology type, staining protocol, preparation protocol), digitization (scanner manufacturer, scanner model, scanning magnification, scanning mode, image file format, color normalization, acquisition device type), and annotation (number of annotators, annotator qualification, inter-annotator agreement, annotation granularity, ground truth basis, classification system).

Each variable was coded as *reported* (information explicitly provided in the dataset's primary publication), *not reported* (no mention of the variable), or *not applicable* (variable does not apply to the dataset type). For staining protocol and inter-annotator agreement, we further distinguished between superficial reporting (e.g., “Papanicolaou staining” without procedural detail, or mention of consensus without a quantitative metric) and substantive reporting (protocol variant specified, or quantitative agreement metric provided).

Extraction was performed by two reviewers independently. A calibration round was conducted on the first five datasets, after which the coding scheme was refined to resolve ambiguities. Disagreements on the remaining datasets were resolved by discussion.

#### 2.2.5 Analysis

Reporting completeness was assessed descriptively. For each variable, we computed the proportion of datasets reporting it. We computed a composite reporting score for each dataset as the count of the 16 key pre-analytical variables reported (range: 0–16). Reporting rates were stratified by dataset source (public vs. private institutional) and by publication period. No formal risk-of-bias assessment was performed, consistent with scoping review methodology (Tricco et al., 2018).

This scoping review was conducted between January 2026 and May 2026, following the Preferred Reporting Items for Systematic Reviews and Meta-Analyses extension for Scoping Reviews (PRISMA-ScR) guidelines (Tricco et al., 2018).

## 3 Results

### 3.1 Study selection and general characteristics

The extraction process yielded 28 cervical cytology datasets from studies published between 2005 and 2025. The majority of studies employed a retrospective design (25/28; 89.3%), with the remainder using a mixed retrospective-prospective approach (3/28; 10.7%). China was the most represented country of data origin (11/28; 39.3%), followed by Brazil (2/28), the United States (2/28), Japan (2/28), and Denmark, Greece, Hungary, India, Nepal, Portugal, France, Australia, Mexico, and Argentina with one dataset each.

In terms of dataset provenance, 13 datasets (46.4%) were publicly available—including well-known benchmarks such as SIPaKMeD (Plissiti et al., 2018), Herlev (Jantzen et al., 2005), CRIC (Rezende et al., 2021), and HiCervix (Cai et al., 2024)—while 10 (35.7%) were private institutional collections, and 4 (14.3%) combined public and private data. The majority were single-center datasets (16/28; 57.1%), while 9 (32.1%) were multi-center. Class distribution was reported with explicit counts in 23 datasets (82.1%), but the number of patients was reported in only 13 (46.4%). Train/validation/test split information was provided in 22 datasets (78.6%).

Regarding model architectures, CNNs were the most common category (15/28; 53.6%), followed by ensemble approaches (3/28), hybrid CNN-ViT models (2/28), traditional machine learning (2/28), and Vision Transformers (1/28). Transfer learning was used in 13 studies (46.4%) and not used in 7 (25.0%), while 5 studies (17.9%) did not report whether transfer learning was employed.

### 3.2 Sample preparation reporting

The cytology preparation type was reported in 24 of 28 datasets (85.7%). Among those reporting, the most common preparation type was liquid-based cytology other than ThinPrep or SurePath (7/28; 25.0%), followed by conventional Pap smear (8/28; 28.6%), ThinPrep (5/28; 17.9%), SurePath (1/28; 3.6%), and mixed preparations (3/28; 10.7%). Four datasets (14.3%) did not specify the cytology type used.

Staining protocol information was provided in 21 datasets (75.0%), but the depth of reporting varied substantially. In the vast majority of cases (19/28; 67.9%), the staining protocol was named only–that is, the study mentioned the use of Papanicolaou staining without specifying the protocol variant (progressive or regressive), reagent details, or immersion durations. Only 2 datasets (7.1%) provided a detailed description of their staining procedure: the CRIC dataset (Rezende et al., 2021) described the use of hematoxylin and two cytoplasmic stains, while the Mendeley LBC dataset (Hussain et al., 2020) specified haematoxylin and eosin staining with procedural details. The remaining 7 datasets (25.0%) made no mention of staining at all.

The sample preparation protocol was similarly documented: 13 datasets (46.4%) named the preparation method without elaborating on it, 6 datasets (21.4%) provided detailed descriptions of their preparation procedures, and 6 datasets (21.4%) did not report the preparation method. In practice, this means that for the majority of the reviewed datasets, a researcher seeking to reproduce the data acquisition pipeline knows only that “Papanicolaou staining was used”—a statement that encompasses a wide family of protocol variants with known inter-laboratory variability, as discussed in Section 2.1.2.

### 3.3 Digitization reporting

The type of image acquisition device was reported in 26 of 28 datasets (92.9%). Among those reporting, the majority used a conventional microscope with a mounted digital camera (18/28; 64.3%), while whole-slide image (WSI) scanners were used in only 7 datasets (25.0%), and 2 datasets used smartphone-based acquisition devices. This finding is notable because microscope-captured images and WSIs differ fundamentally in field of view, resolution uniformity, illumination consistency, and automated focusing capabilities—differences that affect model transferability but are rarely discussed in the context of reported performance metrics.

The scanner or microscope manufacturer was specified in 20 datasets (71.4%), and the specific model was identified in 21 datasets (75.0%). Olympus was the most frequently used manufacturer (6 datasets), followed by Nikon (2), Leica (2), Carl Zeiss (1), and 3DHistech (1); several Chinese datasets used multiple scanner brands. Scanning magnification was reported in 22 datasets (78.6%), with 40 × being the most common (9 datasets), followed by 20 × (4 datasets). Eight datasets reported magnifications outside these standard values, and 6 datasets did not specify magnification at all.

Image resolution was the most consistently reported digitization variable, with 27 of 28 datasets (96.4%) providing pixel dimensions or spatial resolution values. However, three other digitization variables showed substantially lower reporting rates:

- Scanning mode was reported in only 12 of 28 datasets (42.9%). Among those reporting, 7 used single-plane capture and 5 used extended depth of focus (EDF). No dataset in our sample reported using z-stack or volumetric scanning. The remaining 16 datasets (57.1%)—the majority—made no mention of their scanning mode, despite the critical importance of focal plane strategy for cytology image quality, as discussed in Section 2.1.3.
- Image file format was reported in only 11 of 28 datasets (39.3%). Among those specifying a format, JPEG (3 datasets) and PNG (2 datasets) were the most common output formats, with several datasets reporting proprietary WSI formats (SVS, MRXS). The remaining 17 datasets (60.7%) did not document their image format or compression settings.
- Color normalization was the least reported variable in the entire extraction grid, addressed in only 6 of 28 datasets (21.4%). Of these, 4 explicitly stated that no color normalization was applied, and only 2 described the normalization method used. The remaining 22 datasets (78.6%) made no mention of whether color normalization was performed, leaving it unknown whether the input images underwent any standardization of staining appearance prior to model training.

### 3.4 Annotation reporting

Annotator qualification was the most consistently reported annotation variable, identified in 27 of 28 datasets (96.4%). Cytopathologists and pathologists were the most common annotator profiles (7 datasets each), followed by mixed teams of cytopathologists and cytotechnologists (7 datasets) and cytotechnologists alone (3 datasets); the remaining 3 datasets reported other annotator profiles or did not specify them in sufficient detail to assign to one of these categories. The number of annotators was reported in 21 datasets (75.0%), with 3 annotators being the most common configuration (9 datasets), followed by single annotators (4 datasets) and larger teams of 4–6 annotators (5 datasets). One dataset (CCS-127K) involved 23 annotators.

Annotation granularity was documented in 25 datasets (89.3%), with a mixed granularity approach (combining cell-level and slide-level labels) being most frequent (11 datasets), followed by bounding box annotations (5 datasets), pixel-level segmentation masks (3 datasets), cell-level labels (3 datasets), and slide-level labels (2 datasets).

The classification system was reported in 24 datasets (85.7%). The Bethesda System (TBS) was the most commonly used (13 datasets; 46.4%), while 9 datasets (32.1%) used custom classification schemes—typically binary (normal/abnormal) or simplified multi-class categories derived from TBS or CIN grading; the remaining 2 datasets (7.1%) used other classification systems.

Inter-annotator agreement (IAA) was one of the most poorly reported variables. Eleven datasets (39.3%) made no mention of agreement between annotators. Eight datasets (28.6%) mentioned that a consensus or review process was used to resolve disagreements but did not provide a quantitative agreement metric. Only 4 datasets (14.3%) reported a formal IAA metric: BMT_ThinPrep (Welch et al., 2024) required 100% class consensus for inclusion, HiCervix (Cai et al., 2024) used Intersection over Union (IoU > 0.4) for merging bounding boxes, (Kanavati et al., 2022) reported Fleiss' kappa values ranging from 0.568 to 0.861 among 16 cytoscreeners, and RIVA (Perez Bianchi et al., 2025) reported agreement rates of 94% for binary lesion classification and 74% across the full eight-category Bethesda scheme.

Ground truth basis was documented in 24 datasets (85.7%). Cytology consensus among multiple experts was the most common ground truth approach (14 datasets; 50.0%), followed by single expert cytological reading (5 datasets; 17.9%). Only 3 datasets (10.7%) used histopathological confirmation as the ground truth—the approach recommended by Chong and Bychkov (2025) for robust AI validation. Two datasets (7.1%) relied on pre-existing labels from public datasets without re-evaluating or documenting the original labeling process.

### 3.5 Overall reporting completeness

To assess overall reporting quality, we computed a composite reporting score for each dataset based on 16 key pre-analytical variables spanning sample preparation (3 variables), digitization (7 variables), and annotation (6 variables). Each variable was scored as reported (1) or not reported (0), yielding a maximum possible score of 16.

Table 1 presents the composite reporting score for each of the 28 datasets, along with per-category reporting indicators. The mean composite score was 11.4 out of 16 (71.2%), with a median of 12. The highest-scoring dataset was APACC (Kupas et al., 2024) at 15/16, missing only color normalization information. Five additional datasets achieved scores of 14/16: BMT_ThinPrep (Welch et al., 2024), the dataset from (Cheng et al., 2021), DCCL (Zhang et al., 2019), the dataset from Nambu et al. (2022), and RIVA (Perez Bianchi et al., 2025). At the other extreme, BJTUCELL (Wu et al., 2022) reported only 2 of 16 variables, and SIPaKMeD (Plissiti et al., 2018)—one of the most widely used benchmarks in the field—scored only 8/16, lacking documentation of cytology type, preparation protocol, scanning magnification, scanning mode, image format, color normalization, and inter-annotator agreement. Given that SIPaKMeD is used as a benchmark in numerous AI studies (Mbelwa et al., 2026), this documentation gap propagates to every study that relies on it for training or evaluation. Table 2 summarizes the reporting rates for all extracted variables.

**Table 1:** Composite pre-analytical reporting scores for all 28 reviewed datasets, sorted by total score (descending).

Dataset	References	Src	Prep.	Digit.	Annot.	Total
			(/3)	(/7)	(/6)	(/16)
APACC	Kupas et al., 2024	Pub	3	6	6	15
BMT ThinPrep	Welch et al., 2024	Pub	3	5	6	14
Cheng et al.	Cheng et al., 2021	Priv	3	5	6	14
DCCL	Zhang et al., 2019	Priv	3	5	6	14
Nambu et al.	Nambu et al., 2022	Priv	3	5	6	14
RIVA	Perez Bianchi et al., 2025	Pub	2	6	6	14
Cervix93	Phoulady and Mouton, 2018	Pub	3	5	5	13
CPSMI2025	Ocampo-López-Escalera et al., 2025	Pub	2	5	6	13
HMCHH-TCT	Zhang et al., 2025	Pub	2	5	6	13
Kanavati et al.	Kanavati et al., 2022	Priv	3	5	5	13
Mendeley LBC	Hussain et al., 2020	Pub	3	6	4	13
Xiang et al.	Xiang et al., 2020	Priv	3	4	6	13
CRIC	Rezende et al., 2021	Pub	3	3	6	12
EDoF Fraunhofer	Albuquerque et al., 2023	Pub	3	5	4	12
Herlev	Jantzen et al., 2005	Pub	3	3	6	12
ISBI 2014	Lu et al., 2015	Pub	3	5	4	12
CCS-127K	Jiang et al., 2025	Mix	2	3	6	11
HiCervix	Cai et al., 2024	Pub	1	4	6	11
Jia et al.	Jia et al., 2020	Mix	2	4	5	11
CytoCervix	Panta et al., 2025	Mix	2	4	4	10
ISBI 2015	Lu et al., 2017	Pub	3	5	2	10
Pirovano et al.	Pirovano et al., 2021	Mix	3	2	5	10
Araujo et al.	Araújo et al., 2019	Priv	3	4	2	9
CCEDD	Liu et al., 2022	Priv	0	4	5	9
Zhu et al.	Zhu et al., 2021	Priv	3	4	2	9
SIPaKMeD	Plissiti et al., 2018	Pub	1	3	4	8
Wang et al.	Wang et al., 2020	Priv	1	3	4	8
BJTUCELL	Wu et al., 2022	Priv	0	0	2	2
Mean (all datasets)			2.4	4.2	4.8	11.4
Mean reporting rate			78.6%	60.2%	80.4%	71.2%

Variables are aggregated into three categories: Preparation (three variables: cytology type, staining protocol, preparation protocol), Digitization (seven variables: scanner manufacturer, scanner model, scanning magnification, scanning mode, image file format, color normalization, acquisition device type) and Annotation (six variables: number of annotators, annotator qualification, inter-annotator agreement, annotation granularity, ground truth basis, classification system). Each cell shows the number of variables reported out of the category total. Src dataset source (Pub, public; Priv, private institutional; Mix, mixed).

**Table 2:** Reporting rates for pre-analytical variables across 28 cervical cytology datasets.

Category	Variable	Reported	Rate (%)
Sample preparation	Cytology type	24/28	85.7
	Staining protocol (any level)	21/28	75.0
	—of which detailed	2/28	7.1
	Preparation protocol	21/28	75.0
Digitization	Image resolution	27/28	96.4
	Acquisition device type	26/28	92.9
	Scanning magnification	22/28	78.6
	Scanner model	21/28	75.0
	Scanner manufacturer	20/28	71.4
	Scanning mode	12/28	42.9
	Image file format	11/28	39.3
	Color normalization	6/28	21.4
Annotation	Annotator qualification	27/28	96.4
	Annotation granularity	25/28	89.3
	Classification system	24/28	85.7
	Ground truth basis	24/28	85.7
	Number of annotators	21/28	75.0
	Inter-annotator agreement (any level)	14/28	50.0
	—of which with quantitative metric	4/28	14.3
Dataset	Dataset source (public/private)	27/28	96.4
	Single vs. multi-center	26/28	92.9
	Class distribution	23/28	82.1
	Number of patients	13/28	46.4
Reproducibility	Hardware specification	17/28	60.7
	Code availability	16/28	57.1
	Hyperparameter detail	21/28	75.0

Variables are grouped by category and sorted by reporting rate within each category. “Named only” indicates that the variable was mentioned (e.g., “Papanicolaou staining”) without procedural detail.

When stratified by dataset source, publicly available datasets achieved a slightly higher mean reporting score (12.3/16) than private institutional datasets (10.5/16). This difference likely reflects the fact that public datasets are typically accompanied by a dedicated data descriptor publication that includes acquisition details, whereas private datasets are described only within the methods sections of the studies that use them.

We observed a modest improvement in reporting completeness over time: datasets from 2024–2025 achieved a mean score of 12.6/16, compared to 10.5/16 for datasets from 2018–2019 and 10.7/16 for 2020–2021. The 2022 cohort showed a notable dip (mean 9.5/16, driven by several minimally documented datasets), followed by recovery in subsequent years. However, these trends should be interpreted cautiously given the small sample sizes per year.

Table 3 ranks the pre-analytical variables from most to least consistently reported. The three best-reported variables—image resolution (96.4%), annotator qualification (96.4%), and acquisition device type (92.9%)—are those for which the information is intrinsic to describing the dataset or readily available from equipment metadata. In contrast, the three worst-reported variables—color normalization (21.4%), image file format (39.3%), and scanning mode (42.9%)—are precisely those identified in Section 2.1 as significant sources of variability for AI model performance. This pattern suggests a systematic gap: the variables most critical for understanding data acquisition conditions and assessing model transferability are the ones least likely to be documented.

**Table 3:** Pre-analytical variables ranked from most to least consistently reported.

Variable	Reporting rate (%)
Image resolution	96.4
Annotator qualification	96.4
Acquisition device type	92.9
Annotation granularity	89.3
Cytology type	85.7
Ground truth basis	85.7
Classification system	85.7
Scanning magnification	78.6
Staining protocol	75.0
Scanner model	75.0
Number of annotators	75.0
Scanner manufacturer	71.4
IAA (any mention)	50.0
Scanning mode	42.9
Image file format	39.3
Color normalization	21.4
IAA (quantitative metric)	14.3

The four worst-reported variables (below the dashed line) correspond to factors identified in the literature as significant sources of AI model performance variability (IAA = Inter-Annotator Agreement).

## 4 Discussion

### 4.1 Principal findings

This scoping review assessed the extent and quality of pre-analytical reporting across 28 cervical cytology datasets used in AI research. The central finding is a systematic disconnect between what is most important for understanding an AI model's data acquisition context and what is most consistently documented. Variables that are straightforward to state—image resolution (96.4%), annotator qualification (96.4%), acquisition device type (92.9%)—are reported almost universally. In contrast, variables that directly determine whether a model's performance will transfer to a different clinical setting—scanning mode (42.9%), image file format (39.3%), color normalization (21.4%), and quantitative inter-annotator agreement (14.3%)—are documented in fewer than half of the reviewed datasets.

The mean composite reporting score was 11.4 out of 16 possible points (71.2%), indicating that the typical dataset description omits roughly one-third of the pre-analytical information needed to fully characterize its acquisition conditions. Digitization was the weakest reporting category (mean 4.2/7), followed by sample preparation (2.4/3) and annotation (4.8/6). Notably, staining protocol—despite being identified in the digital pathology literature as one of the most significant sources of inter-laboratory color variability (Khan et al., 2026; Tellez et al., 2019)—was described in procedural detail in only 2 of 28 datasets; the remaining 19 datasets that mentioned staining at all did so with a phrase no more specific than “Papanicolaou staining.” We observed a modest improvement in reporting completeness among datasets published in 2024–2025 (mean 12.6/16) compared to earlier years, but the improvement was neither large nor monotonic, suggesting that the field has not yet established a clear upward trajectory in reporting standards.

### 4.2 Implications for reproducibility and cross-study comparison

The most immediate consequence of incomplete pre-analytical reporting is that performance differences across studies become uninterpretable. When two studies both report 97% accuracy on the SIPaKMeD dataset (Plissiti et al., 2018), the comparison tells us something about their model architectures but very little about clinical utility—because SIPaKMeD itself scores only 8/16 on our reporting grid, lacking documentation of its cytology type, preparation protocol, scanning magnification, scanning mode, image format, color normalization, and inter-annotator agreement. Any performance figure reported on this benchmark inherits these documentation gaps.

This problem extends to meta-analyses that aggregate performance metrics across studies. Liu et al. (2025), in the most comprehensive meta-analysis of AI in cervical cytology to date, identified sample size and ground truth definition as significant sources of heterogeneity through regression analysis. However, acquisition-related variables—preparation type, scanner model, scanning mode, staining protocol—were not available as covariates in their regression, precisely because they are inconsistently reported in the primary studies. Our findings provide empirical evidence for why these variables were unavailable: scanning mode was missing in 57.1% of datasets, and color normalization status in 78.6%. The heterogeneity that (Liu et al., 2025) observed but could not fully explain may be partly attributable to these unreported acquisition differences.

The problem is compounded by the dominance of microscope-captured images in the field. We found that 64.3% of reviewed datasets were acquired using a conventional microscope with a mounted digital camera, rather than a whole-slide image scanner. This is a fundamental data modality distinction—microscope-captured images differ from WSIs in field of view, resolution uniformity, illumination consistency, and automated focusing—yet it is rarely discussed when comparing results across studies. A model trained on microscope-captured SIPaKMeD patches and a model trained on WSIs from a commercial scanner are operating on fundamentally different data, even if both are classified as “cervical cytology AI.”

Welch et al. (2024) demonstrated the practical consequence of preparation-method mismatch: deep learning models trained on SurePath images achieved over 90% accuracy on SurePath test data but dropped to 60.29% on ThinPrep images, even with domain adaptation. Our finding that only 85.7% of datasets specify their cytology type—and that the distinction between different LBC methods is sometimes omitted entirely—means that readers of published studies often cannot assess whether such preparation-induced domain shift may affect the claimed results.

### 4.3 Implications for clinical deployment

The reporting gaps we identify have direct consequences for clinical deployment decisions, particularly in low- and middle-income countries (LMICs) where AI-assisted cervical screening holds the greatest potential for impact (Liu et al., 2025; Mbelwa et al., 2026).

Chong and Bychkov (2025) demonstrated that the real-world performance of commercial AI cytology systems can deviate substantially from manufacturer specifications, citing a case where a vendor's claimed specificity of ≥63.5% dropped to 12% in an independent validation study. They attributed this in part to differences in acquisition context—LBC type, scanner, and staining—between the conditions under which the system was developed and those in the validation setting. Our findings indicate that the research literature provides weak support for anticipating such failures: if the studies on which a deployment decision is based do not report their acquisition conditions, there is no way to assess whether the target deployment setting matches the conditions under which the reported performance was achieved.

This concern is particularly acute for LMICs, where acquisition conditions are likely to differ most from those in the predominantly high-resource settings where most AI models are developed and validated. Our data show that 64.3% of datasets used microscope-captured images rather than WSIs—a ratio that may reflect the equipment available in resource-limited settings. However, models trained on WSI data from high-throughput scanners may not generalize to the lower-resolution, less uniform images produced by these microscopes, and vice versa. Mbelwa et al. (2026) note that computer vision methods hold particular promise for low-resource environments due to their scalability and potential for mobile deployment, but this promise depends on the assumption that models will generalize to the acquisition conditions present in those settings—an assumption that current reporting practices make impossible to verify.

### 4.4 Relationship to existing reporting standards

Several reporting guidelines exist for AI studies in medical imaging. TRIPOD+AI, published in 2024 (Collins et al., 2024), provides a 27-item checklist for studies developing or evaluating clinical prediction models, including items on data source description and intended use. The Checklist for Artificial Intelligence in Medical Imaging (CLAIM), originally introduced in 2020 and updated in 2024 (Mongan et al., 2020; Tejani et al., 2024), provides reporting recommendations for AI applications in medical imaging including classification, segmentation, and reconstruction. The CLAIM 2024 update notably added Item 13, which asks authors to provide details about the image acquisition protocol—a recognition that this information had been insufficiently reported in prior work.

These guidelines represent important steps toward standardized reporting, but they address the problem at a general level. TRIPOD+AI recommends describing the data source but does not specify which pre-analytical variables should be reported for cytology vs. radiology vs. dermatology. CLAIM asks for image acquisition details but does not enumerate the specific parameters (preparation type, staining variant, scanning mode, color normalization) that are relevant to cytological analysis. The gap we identify is domain-specific: the five-dye Papanicolaou staining system, the distinction between ThinPrep and SurePath, the three-dimensionality of cytology preparations requiring z-stack or EDF scanning—these are variables that matter specifically for cervical cytology AI and that would not appear in a generic imaging AI checklist.

This parallels the experience of other biomedical fields. The MIQE guidelines (Minimum Information for Publication of Quantitative Real-Time PCR Experiments), published in 2009 (Bustin et al., 2009), demonstrated that domain-specific reporting checklists can have a transformative effect on reproducibility. Before MIQE, qPCR studies routinely omitted critical methodological details—primer sequences, amplification efficiencies, reference gene validation—making results irreproducible even when the underlying experiments were sound. MIQE provided a checklist of essential information that, once adopted by journals, substantially improved the quality and reproducibility of the qPCR literature. The PRECY-AI checklist proposed in Section 5 follows this logic: it translates the domain knowledge of cervical cytology into a concrete set of reporting items tailored to the specific sources of variability that affect AI model performance in this field.

### 4.5 Limitations

This study has several limitations. First, as a scoping review, it provides breadth of coverage rather than depth of individual study assessment; we did not perform a formal risk-of-bias evaluation of the included datasets. Second, our analysis assesses what is *written* in published descriptions, not necessarily what was *done* during data acquisition. It is possible that some studies controlled for pre-analytical variables—such as color normalization or scanning mode—without reporting them, meaning our results may underestimate the actual level of methodological rigor in the field. Third, our extraction was performed on dataset descriptions as they appeared in their primary publications; some datasets may have been described in greater detail in supplementary materials, companion websites, or subsequent publications that we did not capture. Fourth, the sample of 28 datasets, while covering the most widely used public benchmarks and a range of private institutional collections, is not exhaustive; a larger sample might reveal different patterns. Fifth, our study was limited to English-language publications, which may exclude relevant datasets documented in other languages. Finally, we do not assess whether better pre-analytical reporting correlates with better or more generalizable model performance—establishing such a relationship would require a different study design, such as a meta-regression with acquisition variables as predictors of cross-dataset performance.

Finally, while we assessed the PRECY-AI checklist through expert consensus, a formal validation study in clinical environments remains a necessary next step. Regarding programmatic assessment, our current framework focuses primarily on the standardization of retrospective data acquisition reporting rather than educational evaluation tools, though we acknowledge that repetitive feedback loops and regular reflection are vital for the continuous development of AI models. Future reporting standards might also benefit from addressing annotation granularity in greater detail (e.g., distinguishing between single-cell, patch-level, or contextual annotations) and documenting the use of auxiliary modalities that improve clinical sensitivity, such as green filters, Visual Inspection with Acetic Acid (VIA), and Visual Inspection with Lugol's Iodine (VILI).

## 5 PRECY-AI: a proposed minimum reporting checklist

The findings of this review indicate that pre-analytical reporting in cervical cytology AI studies is inconsistent, with the most consequential variables for model transferability being the least frequently documented. Existing reporting guidelines such as TRIPOD+AI (Collins et al., 2024) and CLAIM (Mongan et al., 2020; Tejani et al., 2024) provide valuable general frameworks, but they do not specify which pre-analytical variables are relevant to cytological analysis. To address this domain-specific gap, we propose PRECY-AI (Pre-analytical Reporting Checklist for Cervical Cytology AI), a minimum set of reporting items designed to be completed alongside—not in place of—existing general-purpose guidelines.

### 5.1 Design principles

PRECY-AI was designed around three principles. First, *minimalism*: the checklist includes only variables for which there is evidence—from the digital pathology literature, from our extraction data, or from both—that they affect AI model performance or are necessary for assessing transferability. We deliberately excluded variables that, while scientifically interesting, would impose a reporting burden disproportionate to their informational value. Second, *feasibility*: every item on the checklist is information that researchers either already know (because they performed the data acquisition) or can determine with minimal effort (e.g., by checking scanner specifications or software settings). No item requires additional experimentation. Third, *complementarity*: PRECY-AI is designed to be used alongside TRIPOD+AI or CLAIM, filling the domain-specific gap rather than duplicating their coverage of model architecture, training procedure, and performance evaluation.

The checklist is organized into four categories corresponding to the stages of the data acquisition pipeline described in Section 2.1: sample preparation, digitization, annotation, and dataset characterization. Each item is classified as either Essential (E) or Recommended (R). Essential items are those for which our review found both a low reporting rate (<75%) and strong evidence of impact on AI model performance from the literature. Recommended items are those that are either already well-reported (>90%) but should be maintained, or that provide useful context without being strictly necessary for assessing transferability. Table 4 presents the complete checklist.

**Table 4:** PRECY-AI: Pre-analytical Reporting Checklist for Cervical Cytology AI Studies.

No.	Item	What to report	Level	Current rate
Sample preparation				
1	Cytology preparation type	Conventional Pap smear, ThinPrep, SurePath, or other LBC (specify). If mixed, state the distribution across types.	E	85.7%
2	Staining protocol	At minimum: stain name (Papanicolaou, H&E, other), method type (progressive or regressive), and hematoxylin variant (Harris, Gill's, Mayer's). Ideally: key immersion durations and any deviations from standard protocol.	E	7.1%^a^
3	Preparation protocol	Description of slide preparation procedure or reference to a standard protocol (e.g., manufacturer's protocol for ThinPrep T2000 processor).	R	75.0%
Digitization				
4	Acquisition device type	WSI scanner, microscope with digital camera, or smartphone/portable device.	E	92.9%
5	Scanner/microscope identification	Manufacturer and model name (e.g., “Hamamatsu NanoZoomer S360” or “Olympus BX53 with Excelis HD camera”).	E	75.0%
6	Scanning magnification	Objective magnification used for image capture (e.g., 20 × , 40 × ).	E	78.6%
7	Scanning mode	Single-plane, z-stack (specify number of planes and interval), extended depth of focus, or volumetric. If single-plane, state whether this was a deliberate choice or a scanner limitation.	E	42.9%
8	Image file format and compression	Output format (e.g., SVS, NDPI, TIFF, JPEG, PNG) and, for lossy formats, the compression quality parameter.	R	39.3%
9	Color/stain normalization	State whether any color or stain normalization was applied to images before model training. If yes, specify the method (e.g., Macenko, Reinhard, GAN-based) and reference image selection strategy. If no, state explicitly.	E	21.4%
Annotation				
10	Annotators	Number of annotators and their professional qualification (cytopathologist, pathologist, cytotechnologist, trainee).	E	75.0%
11	Inter-annotator agreement	If >1 annotator: report a quantitative agreement metric (e.g., Cohen's κ, Fleiss' κ, percentage agreement, IoU threshold). State the method used to resolve disagreements (consensus, majority vote, senior expert adjudication).	E	14.3%^b^
12	Ground truth basis	Histopathological confirmation, cytological consensus among multiple experts, or single expert reading. If using a public dataset, state the ground truth basis as documented in the dataset's original publication.	E	85.7%
13	Classification system	Name of the classification system (TBS, Pap classes, CIN) and the specific classes used. If a custom scheme is used (e.g., binary normal/abnormal), define the mapping from the original system.	R	85.7%
Dataset characterization				
14	Sample size: images and patients	Total number of images (or slides) and total number of unique patients. If patient-level information is not available, state this explicitly.	E	46.4%^c^
15	Class distribution	Number of images (or cells, or slides) per diagnostic class.	R	82.1%
16	Data availability	State whether the dataset is publicly available, available on request, or restricted. Provide a URL or repository reference if applicable.	R	57.1%

Each item is classified as Essential (E) or Recommended (R). The “Current reporting rate” column indicates the proportion of datasets in our review that reported this variable. The “What to report” column provides the minimum information expected. This checklist is intended to complement general-purpose reporting guidelines such as TRIPOD+AI and CLAIM, not to replace them.

^a^75.0% of datasets mention staining at some level, but only 7.1% provide sufficient procedural detail (see Section 3.2). The rate shown here reflects the stricter criterion.

^b^50.0% of datasets mention inter-annotator agreement at some level, but only 14.3% provide a quantitative metric. The rate shown reflects the stricter criterion.

^c^96.4% of datasets report image counts, but only 46.4% report patient counts. The rate shown reflects the combined requirement.

### 5.2 Rationale for item selection

#### 5.2.1 Sample preparation

Three items address the preparation stage. *Cytology preparation type* (E) distinguishes conventional Pap smears from LBC methods, and among LBC methods, ThinPrep from SurePath and others. This distinction is classified as essential because (Welch et al., 2024) demonstrated that models trained on one LBC type can lose more than 30 percentage points of accuracy when tested on another, even with domain adaptation. *Staining protocol* (E) is classified as essential because, although 75% of datasets mention staining, only 7.1% provide sufficient detail to characterize the protocol variant (progressive vs. regressive, hematoxylin type, immersion durations). The phrase “Papanicolaou staining” alone is insufficient, as it encompasses a family of protocols with documented inter-laboratory color variability (Clarke and Treanor, 2017; Khan et al., 2026). At minimum, the hematoxylin variant (Harris, Gill's, Mayer's) and the method type (progressive or regressive) should be stated. *Preparation protocol* (R) is recommended rather than essential because, while preparation details affect cell morphology, the most critical information (LBC type) is captured by the first item.

#### 5.2.2 Digitization

Six items address the digitization stage, which our review identified as the weakest reporting category overall (mean 60.2%). *Acquisition device type* (E) distinguishes whole-slide image scanners from microscope-with-camera setups and smartphone-based devices—a fundamental data modality distinction that our review found in 64.3% of datasets but that is rarely discussed in the context of model generalizability. *Scanner or microscope identification* (E) asks for the manufacturer and model, which together determine the optical characteristics (numerical aperture, color calibration, sensor type) that produce device-specific image signatures (Howard et al., 2021; Ardon et al., 2025). *Scanning magnification* (E) is essential because it determines the pixel-level resolution at which biological structures are represented, directly affecting what features a model can learn. *Scanning mode* (E) is classified as essential despite—or rather because of—its 42.9% reporting rate, the second-lowest in our extraction. In cytology, where cell clusters are three-dimensional, the choice between single-plane, z-stack, extended depth of focus, and volumetric scanning determines which cellular features are captured and which are lost (Chong and Bychkov, 2025; Patel et al., 2021). *Image file format and compression* (R) and *color normalization* (E) complete the digitization category. Color normalization is classified as essential because its 21.4% reporting rate was the lowest of any variable in our review, and because it represents a preprocessing decision that directly modifies the pixel values the model receives; at minimum, authors should state whether normalization was applied and, if so, which method was used.

#### 5.2.3 Annotation

Four items address the annotation stage. *Number and qualification of annotators* (E) provides the minimum information needed to assess the human expertise behind the labels. *Inter-annotator agreement* (E) is classified as essential because quantitative IAA was reported in only 14.3% of datasets, yet inter-observer variability in cervical cytology is well-documented (Stoler and Schiffman, 2001) and directly affects the reliability of the ground truth against which models are trained. When multiple annotators are involved, a quantitative metric (e.g., Cohen's or Fleiss' kappa, percentage agreement, IoU threshold) should be provided. *Ground truth basis* (E) asks whether labels are confirmed by histopathology, based on cytological consensus, or derived from a single expert reading—a distinction that (Chong and Bychkov, 2025) identify as critical for AI validation. *Classification system and class definitions* (R) is recommended because most datasets (85.7%) already report this, but we include it to ensure that custom classification schemes (e.g., binary normal/abnormal derived from TBS) are explicitly defined.

#### 5.2.4 Dataset characterization

Three items address dataset-level information. *Number of images and patients* (E) is essential because our review found that while image counts are almost universally reported, patient counts are reported in only 46.4% of datasets. Without patient counts, it is impossible to assess whether images are independent observations or multiple views from the same patients, which affects both the effective sample size and the validity of train/test splitting strategies. *Class distribution* (R) is recommended because it is already well-reported (82.1%) but remains important for interpreting performance metrics, particularly in the presence of class imbalance. *Data availability statement* (R) completes the checklist by encouraging transparency about whether the dataset can be accessed for reproduction or external validation.

### 5.3 Practical considerations for adoption

PRECY-AI is designed to impose minimal additional burden on authors. Most items require only a single sentence or a short phrase—information that researchers already possess from their data acquisition process but often omit from manuscripts. To illustrate: reporting Item 7 (scanning mode) requires writing “Images were captured in a single focal plane” or “Z-stacks of 11 planes at 1 μ m intervals were acquired and fused using extended depth of focus”—a single sentence that transforms an ambiguous dataset description into one that allows readers to assess the three-dimensionality of the captured data.

We envision three modes of adoption. First, *voluntary adoption by authors*: researchers can use PRECY-AI as a self-check when writing the methods section of a cervical cytology AI paper, ensuring that key variables are not inadvertently omitted. Second, *journal-level recommendation*: journals that publish cervical cytology AI research (such as *Journal of Pathology Informatics, Diagnostic Cytopathology*, or *Cancer Cytopathology*) could recommend that authors complete the PRECY-AI checklist as supplementary material alongside the existing CLAIM or TRIPOD+AI checklist. Third, *dataset descriptor publications*: journals that publish dataset papers (such as *Scientific Data* or *Data in Brief* ) could incorporate PRECY-AI items into their data descriptor templates for cervical cytology datasets, ensuring that acquisition conditions are documented at the source rather than relying on downstream studies to reconstruct them.

We emphasize that PRECY-AI is a first version. The checklist should be refined through community feedback, and its items may need to be updated as the field evolves—for example, if new scanning technologies or staining methods emerge, or if computational approaches (such as foundation models pre-trained on diverse multi-scanner data) reduce the sensitivity of models to certain acquisition variables. We welcome discussion on both the content and the adoption pathway of the checklist.

## 6 Conclusion

AI-assisted cervical cytology has reached a stage of methodological maturity, with pooled accuracy and sensitivity exceeding 90% across multiple meta-analyses and at least eight commercial systems now available on the international market. Yet this review reveals a persistent blind spot: the conditions under which these results are produced remain inconsistently documented. Across 28 datasets spanning two decades and 16 countries, we found that scanning mode is unreported in 57.1% of cases, color normalization status in 78.6%, and quantitative inter-annotator agreement in 85.7%. Staining protocol—one of the most significant sources of inter-laboratory variability—is described in procedural detail in only 7.1% of datasets. The variables most critical for assessing whether a model's performance will transfer to a new clinical setting are precisely the ones least likely to be documented.

These gaps matter. Without knowing how data was acquired, prepared, digitized, and labeled, it is impossible to determine whether performance differences across studies reflect genuine advances in model design or merely differences in the underlying data. Meta-analyses that aggregate results across undocumented acquisition conditions carry hidden confounders. Deployment decisions—particularly in low- and middle-income countries, where acquisition conditions may differ most from those in model development settings—rest on evidence whose generalizability cannot be assessed.

The PRECY-AI checklist proposed in this paper offers a practical first step toward closing this gap. Its 16 items require no additional experimentation—only that researchers document decisions they have already made. Adoption by authors as a self-check, by journals as a supplementary requirement, and by dataset publishers as a template component could meaningfully improve the interpretability and reproducibility of cervical cytology AI research. We invite community feedback to refine the checklist and encourage its integration alongside established reporting frameworks such as TRIPOD+AI and CLAIM.

The path from high-performing AI models to reliable clinical screening tools runs through the data acquisition pipeline. Reporting that pipeline transparently is not a methodological luxury—it is a prerequisite for the field's next step.
